# Twelve-Months Follow-up of Supervised Exercise after Percutaneous Transluminal Angioplasty for Intermittent Claudication: A Randomised Clinical Trial

**DOI:** 10.3390/ijerph10115998

**Published:** 2013-11-11

**Authors:** Elisabeth Bø, Jonny Hisdal, Milada Cvancarova, Einar Stranden, Jørgen J. Jørgensen, Gunnar Sandbæk, Ole J. Grøtta, Astrid Bergland

**Affiliations:** 1 Faculty of Health Sciences, Oslo and Akershus University, Pilestredet 46, 0130 Oslo, Norway; E-Mails: milada-cvancarova.smastuen@hioa.no (M.C.); astrid.bergland@hioa.no (A.B.); 2 Faculty of Medicine, University of Oslo, Klaus Torgårdsvei 3, 0372 Oslo, Norway; E-Mails: einar.stranden@medisin.uio.no (E.S.); j.j.jorgensen@medisin.uio.no (J.J.J.); gunnar.sandbaek@medisin.uio.no (G.S.); 3 Section of Vascular Investigations, Oslo Vascular Centre, Oslo University Hospital Aker, Trondheimsveien 235, 0586 Oslo, Norway; E-Mail: jonny.hisdal@medisin.uio.no; 4 Department of Vascular Surgery, Oslo Vascular Centre, Oslo University Hospital Aker, Trondheimsveien 235, 0586 Oslo, Norway; 5 Department of Radiology and Nuclear Medicine, Oslo University Hospital Aker, Trondheimsveien 235, 0586 Oslo, Norway; E-Mail: ole.jorgen.grotta@oslo-universitetssykehus.no

**Keywords:** exercise, PTA, intermittent claudication, follow-up, randomised clinical trial

## Abstract

The aim of this study was to explore the effects during 12 months follow-up of 12 weeks of supervised exercise therapy (SET) after percutaneous transluminal angioplasty (PTA) compared to PTA alone on physical function, limb hemodynamics and health-related quality of life (HRQoL) in patients with intermittent claudication. Fifty patients were randomised to an intervention or a control group. Both groups received usual post-operative care and follow-up measurements at three, six and 12 months after PTA. The intervention group performed 12 weeks of SET after PTA. The control group did not receive any additional follow-up regarding exercise. During the 12 months’ follow-up, the members of the intervention group had significantly better walking distance than the control group. The intervention group had a significantly higher HRQoL score in the physical component score of the SF-36, and the domains of physical function, bodily pain and vitality. For limb hemodynamics, there was a non-significant trend towards better results in the intervention group compared to the control group. Conclusion: SET after PTA yielded statistically significantly better results for walking distance and HRQoL in the intervention group than the control group during the 12 months of follow-up.

## 1. Introduction

Peripheral arterial disease (PAD) is a condition where atherosclerotic plaques build up in the arteries. With time, the size of the plague might increase and narrow the lumen of the arteries, consequently limiting the blood flow distal to the affected arterial segment. This flow limitation may cause pain during activity, which is relieved with rest. These symptoms are called intermittent claudication, and they affect approximately 30% of the patients with PAD. PAD itself is present in approximately 20% of people older than 65 years and it increases with age [1]. Because of its high prevalence, high rate of nonfatal cardiovascular ischemic events, high risk of mortality and reduction of quality of life the consequences of PAD are significant [2]. The treatment aim of PAD is to reduce symptoms, improve quality of life and physical function, and prevent further progression and complications. Treatment strategies include lifestyle changes like smoking cessation, exercise, medication and, if necessary, revascularisation, either endovascular or by surgery [1,3,4].

As there is no definitive cure for PAD, prevention of further progression of the disease is of great importance. Previous research and clinical experience have identified several effective treatment options [5,6,7,8]. However, the best treatment in terms of costs, the intensity and frequency of the intervention, and the length of health professionals’ involvement is not known [9]. The scarce knowledge on enabling patients to be independent and master their own everyday lives including management of a life-long disease, are also important factors as to why research is still needed in this field [10]. 

Authors have suggested the relatively new treatment option of supervised exercise training (SET) after percutaneous transluminal angioplasty (PTA) as an important topic for future research [11,12,13]. The possible advantage of adding SET after PTA is the twofold focus on locally increased blood flow during activity in the treated area and the general effects of exercise, which also influence general risk factor development for further manifestation of cardiovascular disease. However, little is known about the effects of SET after PTA for PAD, particularly for longer-term follow-up. To our knowledge, only two studies on SET following PTA have previously been reported [14,15]. Mazari *et al.* [15] used a three-month training intervention, and reported statistically significant improvements in walking distance at three months for all the studied treatment arms (PTA alone, SET alone and PTA+SET) with the PTA+SET group performing better than either treatment alone. At 12 months of follow-up, this advantage was not sustained. For HRQoL, statistically significant improvements were reported throughout the 12-month follow-up, though with no difference between the three treatment arms. Kruidenier *et al.* [14] used a six-month training intervention, but no further follow-up beyond the end of the intervention was reported. They found an increased walking distance with additional SET after PTA after six months compared to PTA alone. However, no additional improvement in HRQoL was observed. 

Thus, the aims of this study were to explore the effects during one year of 12 weeks of SET (not claudicant specific) after PTA and to compare them with those of PTA alone on physical function, limb hemodynamics and HRQoL in patients with severe claudication. We hypothesised that the group offered SET after PTA would have better results in terms of a positive effect on physical function as well as HRQoL and limb hemodynamics.

## 2. Methods

### 2.1. Study Design

The study was a blinded, prospective, randomised clinical trial with parallel group design. It followed the Consolidated Standards of Reporting Trials (CONSORT) statement criteria for reporting clinical trials [16].

### 2.2. Sample and Sample Size Calculation

Recruitment, interventions and data collection were performed at Oslo University Hospital Aker, Oslo, Norway, between March 2010 and June 2013. Patients eligible for participation in this study were patients selected to undergo PTA due to intermittent claudication (Fontaine stage II) after best medical treatment had failed. Best medical treatment consisted of an urgent request of smoking cessation, appropriate medication for lowering lipids and for diabetes mellitus and hypertension if present, and most important, strongly advise to start or continue exercise. A further requirement was availability to return for hospital-based exercise twice weekly for three months. The exclusion criteria were previous PTA on the same leg during the previous two years, a present unsuccessful attempt at PTA, asymptomatic PAD (Fontaine stage I), critical limb ischemia (Fontaine stage III or IV) and reduced walking ability caused by factors other than PAD (*i.e.*, orthopaedic problems, spinal stenosis, angina pectoris or dyspnoea). The lesions were determined by clinical examination, ABI and ultrasound triplex as a part of the assessment by the vascular specialist. If indication for further investigations, the participants were referred to MRA (or CTA if MRA was contraindicated) to better estimate the options for possible endovascular treatment. 

Sample size calculation was performed based on the primary outcome Six-Minute Walk Test (6MWT). According to Perera *et al.* [17] the number needed per group with 80% power for a between-group comparison of a substantial meaningful change in the 6MWT (50 m, standard deviation 50 m) is 13–20, and of a small clinically meaningful change for the 6MWT (20 m, standard deviation 50 m) is 71–115. These numbers are not based specifically on patients with intermittent claudication, however the symptoms of the latter are quite comparable to mild to moderate mobility deficits. We have calculated that with significant level of 5% and keeping statistical power of 80%, we would need 22 patients in each group so that a difference of 30 m or larger would be statistically significantly different from 50 m (a known threshold).

### 2.3. Ethical Considerations

Approval was obtained from the regional research ethics committee, and written informed consent was obtained from each participant. The study was performed according to the Helsinki Declaration and is registered at ClinicalTrials.gov (NCT01109732). 

### 2.4. Randomisation and Blinding

The participants were stratified according to the treatment site (aortoiliac or femoropopliteal) and randomised into the intervention or control group (ratio 3:2) after the PTA. The ratio 3:2 was chosen with regards to the intervention group’s more demanding effort and therefore possibly a greater drop-out rate in this group. A computer-generated block-randomised list was used together with consecutively numbered and sealed envelopes. The administrative staff prepared the sealed envelopes in advance, and the block size and randomisation list were inaccessible to the project coordinator (E.B.), who enrolled the patients and assigned them to the groups. The assessors were blinded to the group assignment. 

### 2.5. PTA and Post-Operative Care

PTA was performed by a vascular interventional radiologist in accordance with the hospital’s guidelines. Access was gained through puncture of the common femoral artery; retrograde for treatment of lesions in the aortoiliac segment and antegrade for treatment of lesions in the femoropopliteal segment. A six French sheath was introduced. Lesions in iliac arteries were all treated with stents primarily. In femoropopliteal lesions we preformed balloon angioplasty, and implanted stent only in case of flow-limiting dissection or significant residual stenosis. Both groups received post-operative care in agreement with the ward’s usual procedures and were discharged either the same day or on the first post-operative day. The discharging doctor and the responsible nurse gave general advice on the importance of exercise, smoking cessation and diet. 

### 2.6. Intervention

The intervention group received hospital-based SET two days per week for 12 weeks. In addition, the participants conducted one home-based exercise session every week. After the period of hospital-based SET, the participants conducted three home-based exercise sessions every week for an additional 12 weeks. 

The SET was based on The Norwegian Ulleval Model [18], a modified cardiac rehabilitation program, and was slightly adjusted to be applicable to this patient group. Each SET session lasted for 60 min and consisted of warm-up exercises, three high-intensity intervals (each lasting for five to ten minutes), two moderate-intensity intervals (each lasting for five to ten minutes) and cool-down exercises, including stretching. The exercises were simple aerobic dance movements and walking, and involved the use of both upper and lower extremities. During walking the participants walked alternating in a circle in the gym, in the corridor or stair climbing. The instructor walked the opposite direction within the circle or close by in the corridor and the stairs to monitor the participants. The exercise intensity was adjusted using the Borg scale of perceived exertion [19] and the beats per minute of the music [18]. During the high-intensity exercises, the participants were motivated to gradually increase their exercise intensity towards 15–17 on the Borg scale, and during the exercise sessions, the patients informed the instructor of their Borg Scale ratings. The participants also used this scale to monitor the home-based exercise session each week. No extra equipment was required for this program. Each session had between two and twelve participants. The control group did not receive any additional follow-up regarding exercise beyond general advice on the importance of exercise at discharge. 

### 2.7. Assessments at Baseline and Follow-ups

All measurements were taken during a single visit at baseline (prior to the planned PTA) and three, six and 12 months after the PTA. 

The primary outcome was a standardised Six-Minute Walk Test (6MWT). The 6MWT was performed in a 30 m pre-marked hospital corridor, and instructions and encouragements were given in accordance with the test’s guidelines [20]. This test is well validated in PAD patients and has shown good reliability in this patient group [21,22].

Secondary outcomes were measurement of physical function, limb hemodynamics and HRQoL. The physical function measurements were pain-free walking distance (PFWD) and maximal walking distance (MWD) on a treadmill (graded protocol, 3.2 km/h constant speed, starting with a 0% incline that increased 2% every two minutes up to 10%) [23]. Treadmill testing is a well-accepted means of testing walking distance for this patient group [4,24] and has shown very high reliability [25]. Limb hemodynamics were measured using the ankle-brachial-index (ABI) (ankle-pressure/arm-pressure) by doppler and pulse volume recording (PVR) on the leg by a pressurized cuff on the leg connected to a plethysmograph (Stranden macrolab, Oslo, Norway). In addition, all participants were measured by triplex ultrasound at baseline and all follow-ups. HRQoL was measured with a generic instrument, the Short Form 36 (SF-36) [26], as well as a disease-specific instrument, the Claudication Scale (CLAU-S) [27]. The SF-36 has previously been used in numerous PAD studies and is recommended as one of the most appropriate generic instruments for this patient group with regard to validity, reliability and responsiveness [28,29]. The eight domains on the SF-36 are physical function, physical role, bodily pain, general health, vitality, social function, emotional role and mental health. The SF-36 raw scores were coded and recalibrated following standard guidelines [26], and the items were then summed and transformed into the eight scales ranging from 0 to 100 (higher scores indicate better quality of life). CLAU-S is a valid instrument [30] and has five subscales: daily life, pain, social life, disease-specific anxiety and psychological well-being. The CLAU-S raw scores were also coded, recalibrated, summed and transformed into the five scales ranging from 0–100 (higher scores indicate better quality of life). 

### 2.8. Statistical Analysis

Continuous data in the tables are described with mean and standard deviation or standard error of the mean (SEM) when normally distributed or with median and range when having a skewed distribution. Categorical variables are presented as numbers and percentages. Crude differences between pairs of categorical variables were assessed with Chi-square tests and with Mann-Whitney Wilcoxen test for continuous variables. Changes over time and differences between groups were analysed using mixed models for repeated measures with group, time and the interaction between time and group being modelled as fixed effects. The dependencies between time points were modelled using diagonal covariance matrix. *p*-values ≤ 0.05 were considered statistically significant and all tests were two-sided. All analyses were conducted using SPSS 20.0 (SPSS Corporation, Chicago, IL, USA). 

## 3. Results

**Figure 1:**
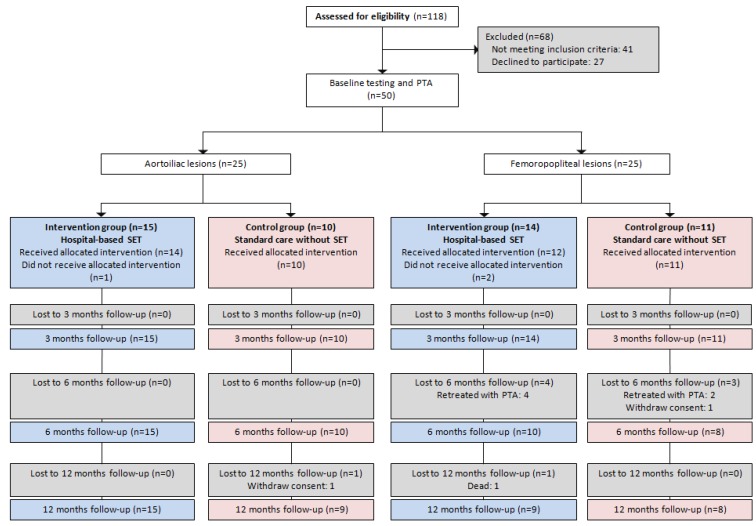
CONSORT flow chart of the study.

Of the 118 patients potentially eligible for the study, 50 participants were included. Participants who did not meet the inclusion criteria were excluded. The main exclusion reasons were work-related obligations, previous PTA during the previous two years, reduced walking ability due to other factors than PAD and lack of interest to participate in the study. Figure 1 shows the flow of participants through the study. Altogether, six participants underwent re-intervention after three months’ follow-up. Two participants withdrew during follow-up, after three and six months, respectively. One participant died before 12 months follow-up. There were no statistically significant differences between genders at baseline regarding the variables connected to the main outcome. The general participant characteristics are shown in Table 1. 

**Table 1:** Participant characteristics at baseline.

	Intervention group (n = 29)		Control group (n = 21)	
	Mean	(SD)	Mean	(SD)
	or		or	
	%	(n)	%	(n)
**Demographics**				
Age (years)	66.9	(7.1)	66	(8.3)
Body mass index (kg/m^2^)	27.2	(5)	27.4	(4)
Gender (men)	14	(48.3)	10	(47.6)
Marital status (married)	18	(62.1)	10	(47.6)
Years of school (>9 years)	22	(75.9)	16	(76.2)
**Blood status**				
Total cholesterol (mmol/L)	4.8	(1)	4.9	(1.1)
HDL ^1^ (mmol/L)	1.7	(0.6)	1.6	(0.6)
LDL ^1^ (mmol/L)	2.6	(0.7)	2.6	(0.8)
Triglycerides (mmol/L)	1.3	(0.6)	1.5	(1)
HbA1c ^1^ (%)	5.9	(0.7)	6.2	(0.6)
**Smoking status**				
Have never smoked	0	(0)	4.8	(1)
Used to smoke	62.1	(18)	52.4	(11)
Currently smoke	37.9	(11)	42.8	(9)
**Current medication**				
Statins	96.6	(28)	90.5	(19)
Platelet inhibitors	86.2	(25)	95.2	(20)
Anticoagulants	6.9	(2)	0	(0)
Hypertension	51.7	(15)	61.9	(13)
**Comorbidity**				
Diabetes	10.3	(4)	19.0	(4)
COPD	6.9	(2)	4.8	(1)
**Previous cardiovascular events**				
Myocardial infarction	27.6	(8)	38.1	(8)
Stroke/TIA ^1^	3.4	(1)	0	(0)
Peripheral arterial surgery or endovascular treatment	17.2	(5)	19.0	(4)

^1^ HDL = high-density lipoproteins; LDL = low-density lipoproteins; HbA1c = hemoglobin A1c; COPD = chronic obstructive pulmonary disease; TIA = transient ischemic attack.

### 3.1. Physical Function

**Figure 2:**
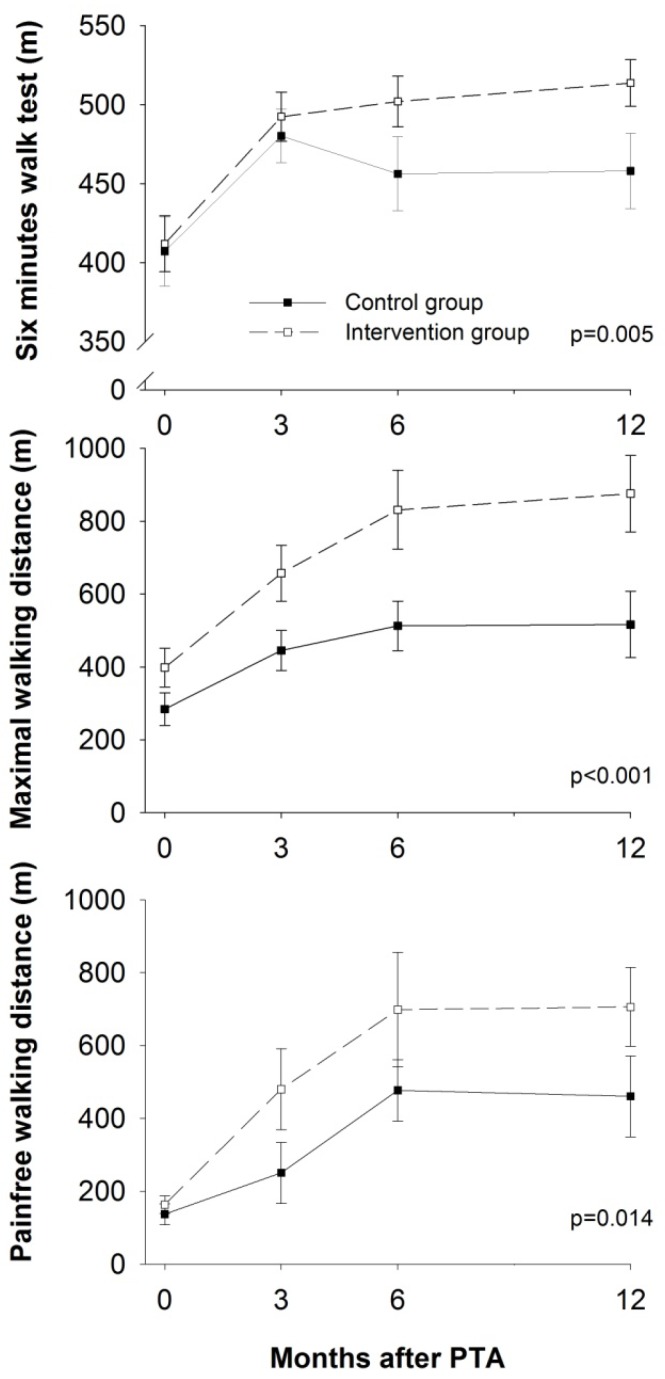
Walking distance. Values are mean, error bars indicate ±SEM. m = metres.

Physical function measured by walking distance (6MWT, MWD and PFWD) showed a statistically significant difference between the two groups at the 12-month follow-up (*p* = 0.005, *p* < 0.001, *p* = 0.014, respectively). The intervention group showed a greater change in walking distance than the control group (Table S1 and Figure 2). In the 6MWT, both groups achieved a statistically significant increase in walking distance at three months’ follow-up. The intervention group continued to increase in walking distance up to 12 months’ follow-up, while the control group showed a decrease in walking distance from three to six months and then maintained this level at 12 months. For the MWD and PFWD, the progress followed the same pattern in both groups: an increase up to six months and maintaining the same level from six to 12 months. When analysed as percentage mean change from baseline to 12 months, the mean change of 6MWD was 23% and 15% in the intervention group and the control group, respectively. For the MWD the percentage mean change from baseline was 107% in the intervention group and 96% in the control group, and for the PFWD the percentage mean change from baseline was 346% in the intervention group and 293% in the control group. 

### 3.2. Limb Hemodynamics

**Figure 3:**
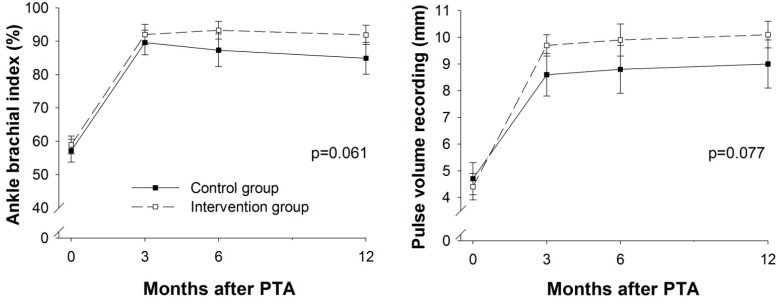
Limb hemodynamics. Values are mean, error bars indicate ±SEM. mm = millimetres.

Regarding the limb hemodynamics, as measured by the ABI and PVR, both groups significantly increased from baseline to three months, which was expected due to the nature of the endovascular treatment. For both the ABI and the PVR, there was a statistical trend (*p* < 0.10) towards better results in the intervention group compared to the control group, but the results were not statistically significantly different between the two groups during the 12 months of follow-up (*p* = 0.061 and *p* = 0.077, respectively) (Figure 3). 

### 3.3. HRQoL

**Figure 4:**
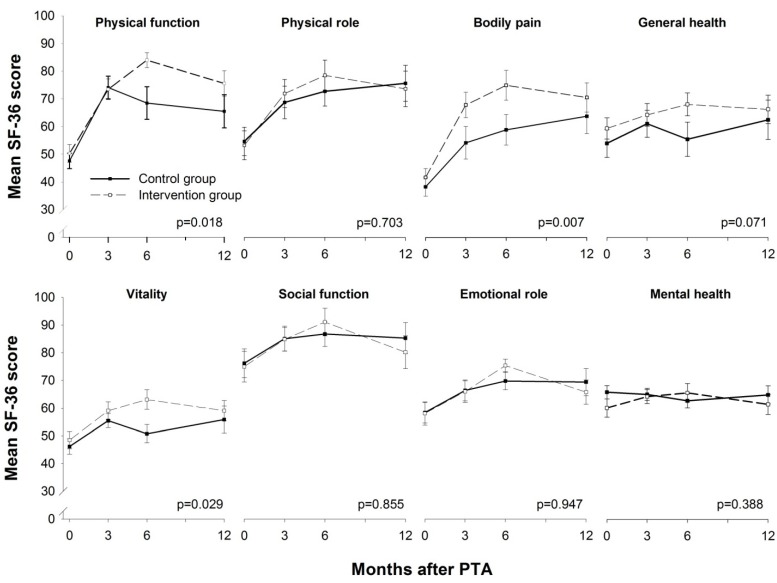
SF-36 domain scores. A score of 0 represents the worst possible health, and 100 represents the best possible health. SF-36 = Short Form-36 Health Survey. Values are mean, error bars indicate ±SEM.

**Figure 5:**
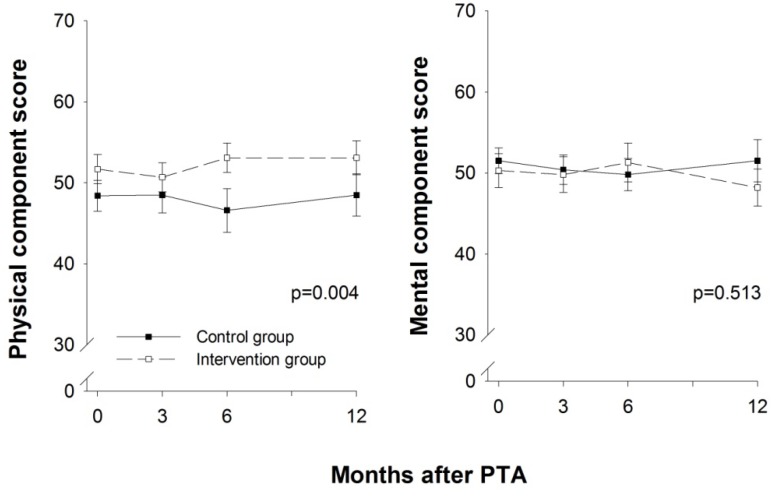
SF-36 component scores. A score of 0 represents the worst possible health, and 100 represents the best possible health. SF-36 = Short Form-36 Health Survey. Values are mean, error bars indicate ±SEM.

HRQoL measured using SF-36 showed a statistically significant difference between the two groups during the 12 months of follow-up for the domains physical function (*p* = 0.018), bodily pain (*p* = 0.007) and vitality (*p* = 0.029) (Figure 4). The same was true of the SF-36 physical component score (*p* = 0.004) (Figure 5), in contrast to the lack of a statistically significant difference in the SF-36 mental component score (*p* = 0.513). The domains that had statistically significant differences between the groups were also the ones with the lowest scores at baseline. The remaining domains showed no statistically significant difference between the groups during the follow-up. 

**Figure 6:**
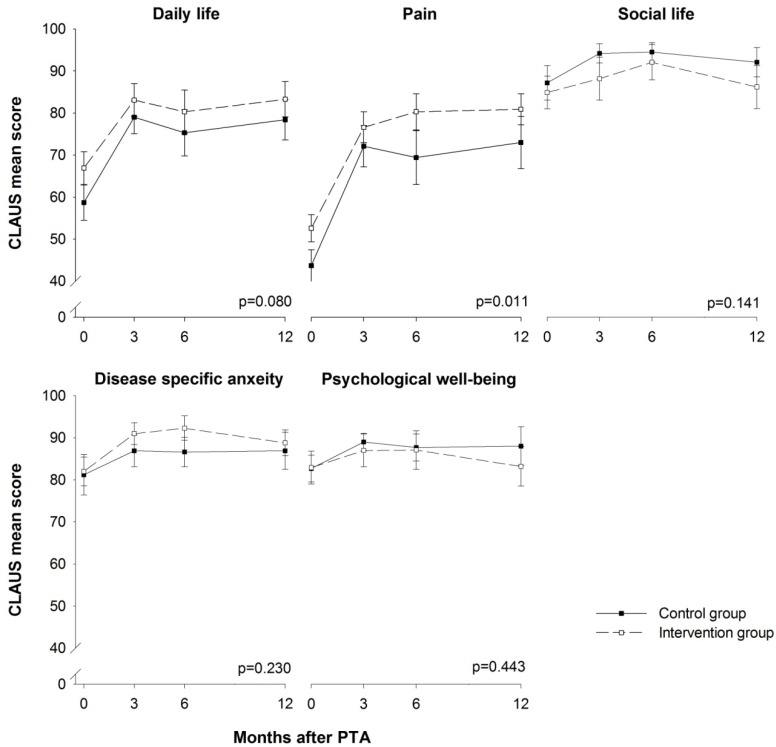
CLAU-S domain scores. A score of 0 represents the worst possible health, and 100 represents the best possible health. CLAU-S = the Claudication Scale. Values are mean, error bars indicate ±SEM.

The pain score of CLAU-S was statistically significantly different between the two groups during the 12 months of follow-up (*p* = 0.011). Regarding the daily life domain, there was a statistical trend (*p* < 0.01) towards a statistically significant difference (*p* = 0.080). The remaining three domains (social life, disease specific anxiety and psychological well-being) did not show any significant difference during the 12 months of follow-up (*p* = 0.141–0.443) (Figure 6). Regarding the results of the CLAU-S, most domains showed a ceiling effect (>20% scored the highest possible score) [31] at follow-up. The exceptions were the domains pain at three months, daily life at 12 months and psychological well-being at three, six and 12 months. The social life domain showed a ceiling effect at baseline and all three follow-ups.

### 3.4. Medication and Adverse Events

There were only minor changes of medication during follow-up. No major adverse events associated with the prescribed follow-up were observed. 

## 4. Discussion

The main finding in the present study was significantly better walking distance in the intervention group than for the control group during the 12 months of the study. With regards to HRQoL, the intervention group had a significantly better score in the physical component of the SF-36, which also mirrors their significantly better results of the physical function, bodily pain and vitality domains. For limb hemodynamics, there was a trend towards better results in the intervention group compared to the control group; however, the results were not statistically significantly different between the two groups during the 12 months of follow-up. 

A statistically significant improvement from baseline has been reported for walking distance, limb hemodynamics and HRQoL at three months follow-up for the present study population, with no statistically significant difference between the two groups [32]. From three to 12 months, the differences for most of the outcomes increased between the groups. At three months, it might be reasonable to think that both groups were strongly influenced by the initial known effect of PTA, more than any additional effect of SET. However, from three months onwards, as the differences increased, the hypothesised effects of SET after PTA seemed to come to pass, and this effect remained even at 12 months. Differences in HRQoL seem to develop later after exercise interventions compared to other outcomes, specifically more physical outcomes such as walking distance and limb hemodynamics [33,34]. In the present study the inter-group differences in HRQoL were greater after three months than at three months, but even so, HRQoL showed less improvement than walking distance and limb hemodynamics. 

Determining clinical significance is difficult for patients with intermittent claudication as the disease impact their HRQoL differently. However, a study on older adults with mild to moderate mobility deficits [17] has a suggested recommendation for a substantial clinical meaningful change for the 6MWT of 50 m and a small clinical meaningful change for the 6MWT of 20 m. These numbers are not based specifically on patients with intermittent claudication, however the symptoms of the latter are quite comparable to mild to moderate mobility deficits. In the present study the mean change from baseline to 12 months of the 6MWT were 97m and 65m for the intervention group and the control group, respectively, and therefore most likely clinically significant for both groups. In addition, the mean changes from baseline to 12 months in the treadmill measures of walking distance were even greater, which strengthens the picture of the clinical significance of this intervention. When analysed with regards to percentage change, the mean change from baseline to 12 months was about 100% for both MWD and PFWD in both groups. Unfortunately, no minimal clinically important difference of change for neither maximal- nor pain-free walking distance on treadmill has been established for patients with intermittent claudication [35] so clear comparisons were not possible. 

The present study’s results differ somewhat from Mazari *et al.* [15] and Kruidenier *et al.* [14]. Mazari *et al.* found PTA+SET to be more effective at three months’ follow-up than PTA or SET alone, but this effect was not sustained at 12 months. Possible explanations for this disparity between the studies might be the use of different treadmills and exercise protocols. The present study had a treadmill protocol with a time-cap of 30 minutes (1,600 m), whereas Mazari *et al.* used a treadmill protocol of a maximum of five minutes (215 m), which most likely did not cover the expected progress of these participants after PTA. The exercise protocol in the present study was a generic, not claudicant-specific, protocol, unlike the protocol of Mazari, which was claudicant specific. In addition, Mazari *et al.* did not report on the patients’ exercise after three months. We strongly encouraged the participants to perform further home-based exercise for another three months after the three months of hospital-based SET sessions. 

Kruidenier *et al.* reported better results at three months’ follow-up compared to the present study. The good results for walking distance after SET+PTA were maintained at six months’ follow-up, but they did no further follow-up beyond six months, so whether the results were sustained is unknown. Compared to the present study, the development from baseline to three months was different, but the results were quite similar at six months. Kruidenier *et al.* observed no difference in HRQoL between the groups at three or six months. In contrast, we observed significant differences between the groups with regards to three domains and the physical component score during the 12 months of follow-up. The exercise protocol of Kruidenier *et al.* was claudicant specific such as that of Mazari *et al.* The exercise intervention in Kruidenier *et al.* was community-based, as opposed to the present exercise intervention, which was hospital-based. The community-based setting, however, might be a more realistic option than a hospital-based intervention because, for instance, transportation costs may be reduced.

We want to point out one specific result in the present study; the possible increased results of the intervention after cessation of the hospital-based SET. Participants in the intervention group increased particularly their walking distance, and they maintained and even somewhat improved their limb-hemodynamics parameters. In terms of HRQoL, we also observed the same trend. Limited data exist on how to maintain an achieved exercise behaviour, and, the most efficient means of action are inconclusive [10]. Potential reasons for the results of the present study may be that participants in the intervention group incorporated new or better habits of exercise and physical activity into their daily life routine during the first three months and continued, as they were encourage to do for the next three months in particular. They may also have felt more secure with regards to activities they could and should do safely by supervision they received during the first three months [36]. In addition, they may have felt and observed the effect of the effort they put into the exercise over the weeks that the intervention lasted and become inspired to continue. The exercises of the intervention were deliberately simple and easy to transfer to other settings for further usage. 

Our study has limitations that need to be addressed. A great portion of the CLAU-S domains showed a ceiling effect early in the follow-up, and one domain even did at baseline, and this outcome measure did not capture the possible progress of the HRQoL in these participants. The exercise intervention was hospital-based, which may limit its generalisability. The sample size was small, however, the majority of our results reached the level of statistical significance. Due to limited sample size we have not had acceptable statistical power to do separate analysis based on treatment level with sufficient precision in the estimate. Therefore, we chose to stratify the participants by the level of the obstruction before randomization into the two groups to be sure that the groups were as similar as possible at baseline. The results of this study do not elucidate the potential difference in the effect of SET after PTA at the aortoiliac- or femoropopliteal level. Our results may therefore be viewed as preliminary with regards to this issue, and should be focused on in future studies. The strength of our study is its design and its inclusion of the trajectories of several measures of physical function, HRQoL and limb hemodynamics. 

## 5. Conclusions

In this study, SET after PTA for intermittent claudication yielded statistically significantly better results during 12 months’ follow-up on walking distance and the more physical components of HRQoL compared to PTA alone. In addition, there was a trend towards better results for limb-hemodynamics in the intervention group. These findings are an important contribution to the evidence-based knowledge of efficient treatment for intermittent claudication, particularly the emerging data on the effect of offering SET after PTA. However, this is based on a small sample size and therefore our results should be interpreted with caution and confirmed in future research. Future research should also include longer follow-up to be able to observe more long term benefits of SET. In addition, we recommend including health-economic analyses to plan the appropriate treatments and evaluate treatment efficacy, as limited economic means is a restricting factor for health services in general, and for non-life-threatening conditions in particular. 

## Supplementary Files

Supplementary File 1 Supplementary Information (PDF, 120 KB)
