# Supplementary material for: Twelve-Months Follow-up of Supervised Exercise after Percutaneous Transluminal Angioplasty for Intermittent Claudication: A Randomised Clinical Trial
Source: Int J Environ Res Public Health. 2013 Nov 11;10(11):5998–6014. doi: 10.3390/ijerph10115998 (PMC3863883; doi:10.3390/ijerph10115998)
Supplement: Supplementary File 1 — Supplementary Information (PDF, 120 KB) [file ijerph-10-05998-s001.pdf]

Twelve-Months Follow-up of Supervised Exercise after Percutaneous Transluminal Angioplasty for Intermittent Claudication: A Randomised Clinical Trial

Table S1. Observed baseline and follow-up measurements.

	Sample size	Mean value at baseline (95% CI)	Mean value at 3 months (95% CI)	Mean value at 6 months (95% CI)	Mean value at 12 months (95% CI)	Mean change: 12 months minus baseline (95% CI)
**Physical function**						
**6 MWT (m)**						
Intervention group	28	412 (375.7–448.3)	492.4 (460.6–524.2)	502.1 (468.9–535.4)	513.7 (482.9–544.5)	96.8 (68–125.7)
Control group	21	407.2 (362.5–453)	480.2 (445.1–515.2)	456.3 (406.8–505.8)	458.1 (407.8–508.3)	64.2 (20.1–108.4)
**MWD treadmill (m)**						
Intervention group	26	398.6 (289.3–507.8)	657.5 (499.2–815.8)	831.8 (604.3–1059.3)	876 (654.9–1097.1)	429.5 (233.7–625.4)
Control group	21	284.6 (191.5–377.6)	445.5 (330.8–560.1)	513.1 (368.7–657.5)	516.9 (324.5–709.4)	274.7 (116.6–432.9)
**PFWD treadmill (m)**						
Intervention group	24	163.5 (113–214)	480.2 (242.5–717.9)	698.4 (355.8–1041)	705.9 (475.8–936)	566.7 (344.3–789)
Control group	20	137.3 (77.5–197.1)	250.6 (61–440.3)	476.9 (297.4–656.4)	460.9 (223.8–698)	402.3 (168.4–636.2)
**Limb hemodynamics**						
**Ankle Brachial Index (%)**						
Intervention group	27	58.9 (53.2–64.6)	92 (85.7–98.2)	93.3 (87.8–98.9)	91.9 (85.8–97.9)	31.4 (23.8–39)
Control group	20	57.2 (50–64.3)	89.6 (81.9–97.2)	87.3 (76.7–97.7)	84.9 (74.7–95)	29.8 (20.4–39.2)
**Pulse Volume Registration (mm)**						
Intervention group	27	4.4 (3.5–5.4)	9.7 (8.8–10.6)	9.9 (8.7–11)	10.1 (9–11.1)	5.9 (4.7–7.2)
Control group	20	4.7 (3.5–5.8)	8.6 (7–10.2)	8.8 (7–10.7)	9 (7.2–10.8)	4.5 (3.3–5.8)
**HRQoL**						
**SF 36**						

Table S1. *Cont.*

	Sample size	Mean value at baseline (95% CI)	Mean value at 3 months (95% CI)	Mean value at 6 months (95% CI)	Mean value at 12 months (95% CI)	Mean change: 12 months minus baseline (95% CI)
**Physical component score**						
Intervention group	25	51.7 (48.1–55.4)	50.7 (47.1–54.4)	53.1 (49.4–56.9)	53.1 (48.6–57.5)	−0.1 (−4.6–4.5)
Control group	21	48.4 (44.5–52.4)	48.5 (44–49.2)	46.6 (40.8–52.3)	48.5 (43.1–54)	1.7 (−2.7–6.1)
**Mental component score**						
Intervention group	25	50.3 (46.1–54.6)	49.2 (45.2–54.4)	51.3 (46.4–56.2)	48.2 (43.5–53)	−1 (−5.6–3.6)
Control group	21	51.5 (48.2–54.7)	50.4 (46.6–54.3)	49.8 (45.6–53.9)	51.5 (46–57)	−1.1 (−5.6–3.4)
**Physical function (PF)**						
Intervention group	29	50.3 (43.7–56.8)	73.5 (65.9–81.1)	84 (78.4–89.7)	75.6 (66.1–85.2)	25.4 (16.4–34.5)
Control group	21	47.6 (41.8–53.5)	74.1 (65.6–82.6)	68.5 (56.1–80.9)	65.5 (52.9–78.1)	19 (7.6–30.4)
**Physical role (RP)**						
Intervention group	26	53.3 (42.6–64)	72 (61.5–82.5)	78.6 (67.1–90.1)	73.7 (60.5–86.9)	20.9 (4.9–36.9)
Control group	21	54.8 (44.2–65.3)	68.8 (56.6–81)	72.8 (61.6–84)	75.7 (62–89.5)	23.5 (13.7–33.3)
**Bodily pain (BP)**						
Intervention group	28	41.7 (35.1–48.2)	67.9 (58.4–77.3)	75 (63.7–86.2)	70.6 (59.5–81.6)	24.9 (14.9–34.9)
Control group	21	38.3 (31.2–45.4)	54.2 (42–66.4)	58.9 (47.3–70.6)	63.8 (50.5–77.1)	27.5 (14.5–40.6)
**General health (GH)**						
Intervention group	27	59.4 (51.5–67.3)	64.3 (55.9–72.7)	68.1 (59.6–76.7)	66.3 (55.6–76.9)	8 (−1.4–17.3)
Control group	21	54 (43.4–64.7)	61.1 (51–71.2)	55.5 (42.3–68.6)	62.5 (47.4–77.6)	10.7 (1.2–20.2)
**Vitality (VT)**						
Intervention group	27	48.5 (42.2–54.8)	59.1 (52.6–65.6)	63.1 (55.7–70.5)	59.1 (51.6–66.6)	10.5 (3.5–17.4)
Control group	21	46.2 (40.3–52.1)	55.5 (50.3–60.7)	50.8 (43.9–57.7)	55.9 (45.5–66.4)	10.6 (2.9–18.3)
**Social function (SF)**						
Intervention group	29	75 (63.8–86.2)	84.9 (76.3–93.6)	91.1 (80.7–101.4)	80.2 (68–92.5)	6.8 (−5.2–18.7)
Control group	21	76.2 (65.3–87.1)	85.1 (75.8–94.4)	86.8 (77.3–96.3)	85.3 (73.4–97.2)	5.9 (−4.6–16.3)

Table S1. *Cont.*

	Sample size	Mean value at baseline (95% CI)	Mean value at 3 months (95% CI)	Mean value at 6 months (95% CI)	Mean value at 12 months (95% CI)	Mean change: 12 months minus baseline (95% CI)
**Emotional role (RE)**						
Intervention group	28	58.1 (49.4–66.8)	66 (58.2–73.8)	75.4 (70.5–80.3)	65.8 (56.7–75)	7.8 (−1.9–17.6)
Control group	21	58.4 (50.6–66.2)	66.4 (67.8–73.3)	69.8 (63–76.6)	69.4 (59.1–79.7)	10.2 (1.2–19.2)
**Mental health (MH)**						
Intervention group	27	60.2 (53.3–67)	64.3 (59.1–69.5)	65.7 (58.8–72.6)	61.5 (54–68.9)	2.2 (−4.7–9)
Control group	21	65.9 (61–70.8)	65.1 (65.1–68)	62.8 (57.3–68.4)	64.9 (58–71.9)	−2.1 (−7–2.7)
**CLAU-S**						
**Daily life (DL)**						
Intervention group	28	66.9 (59–74.9)	83.1 (75.1–91.1)	80.3 (69.5–91)	83.3 (74.7–92)	14.7 (7–22.3)
Control group	21	58.7 (850.1–67.4)	79 (71–87)	75.3 (63.7–87)	78.4 (68.3–88.5)	22.6 (10.3–35)
**Pain (P)**						
Intervention group	28	52.6 (46.1–59.2)	76.6 (68.9–84.3)	80.3 (71.4–89.3)	80.9 (73.3–88.5)	26.1 (16.8–35.4)
Control group	21	43.7 (35.7–51.7)	72.1 (61.9–82.3)	69.5 (55.8–82.9)	73 (59.8–86.1)	29 (15.8–42.2)
**Social life (SL)**						
Intervention group	26	84.9 (76.9–92.8)	88.2 (77.7–98.8)	92.1 (83.4–100.7)	86.2 (75.6–96.8)	1.7 (−6–9.4)
Control group	21	87.2 (78.6–95.8)	94.2 (89.5–98.8)	94.5 (89.6–99.4)	92.1 (84.6–99.5)	4.6 (−1.5–10.6)
**Disease-specific anxiety (DSA)**						
Intervention group	26	82 (75–88.9)	91 (85.7–96.3)	92.3 (886.2–98.4)	88.8 (82.4–95.2)	5.5 (1.1–9.9)
Control group	21	81.2 (71.3–91.1)	86.9 (79.1–94.7)	86.6 (79.2–94.1)	86.9 (77.4–96.4)	5.8 (−8.4–20.1)
**Psychological well-being (PWB)**						
Intervention group	27	81.9 (74–89.8)	87 (79–95)	87.1 (77.5–96.6)	83.2 (73.5–92.8)	2.7 (−4.1–9.5)
Control group	21	82.7 (76–89.3)	89 (84.6–93.4)	87.7 (80.8–94.6)	88 (78.3–97.7)	3.4 (−2.3–9.1)

© 2013 by the authors; licensee MDPI, Basel, Switzerland. This article is an open access article distributed under the terms and conditions of the Creative Commons Attribution license (http://creativecommons.org/licenses/by/3.0/).
